# Time in a Bottle: The Evolutionary Fate of Species Discrimination in Sibling *Drosophila* Species

**DOI:** 10.1371/journal.pone.0031759

**Published:** 2012-02-27

**Authors:** Erin M. Myers, W. Anthony Frankino

**Affiliations:** Department of Biology and Biochemistry, University of Houston, Houston, Texas, United States of America; University of Arkanas, United States of America

## Abstract

Disadvantageous hybridization favors the evolution of prezygotic isolating behaviors, generating a geographic pattern of interspecific mate discrimination where members of different species drawn from sympatric populations exhibit stronger preference for members of their own species than do individuals drawn from allopatric populations. Geographic shifts in species' boundaries can relax local selection against hybridization; under such scenarios the fate of enhanced species preference is unknown. Lineages established from populations in the region of sympatry that have been maintained as single-species laboratory cultures represent cases where allopatry has been produced experimentally. Using such cultures dating from the 1950s, we assess how *Drosophila pseudoobscura* and *D. persimilis* mate preferences respond to relaxed natural selection against hybridization. We found that the propensity to hybridize generally declines with increasing time in experimental allopatry, suggesting that maintaining enhanced preference for conspecifics may be costly. However, our data also suggest a strong role for drift in determining mating preferences once secondary allopatry has been established. Finally, we discuss the interplay between populations in establishing the presence or absence of patterns consistent with reinforcement.

## Introduction

Reinforcement, the process by which natural selection against hybridization strengthens prezygotic reproductive isolation between species, enhances prezygotic isolating mechanisms including interspecific mate discrimination among sympatric taxa [1]–[6]. Reinforcement generates a geographic pattern in species discrimination where members of potentially hybridizing species from sympatric populations exhibit a lower propensity to mate interspecifically than do individuals drawn from allopatric populations [7]–[10]. Although enhanced species discrimination is beneficial in light of natural selection against hybridization [4], its evolutionary fate in the absence of interspecific interactions is not clear. In part, the outcome may hinge on what prevents enhanced species discrimination from initially spreading throughout a species' range. Below, we address two of the most likely possibilities and outline how these generate specific predictions regarding the evolutionary response to relaxation of selection against hybridization.

One reason why enhanced species discrimination may remain restricted to regions of sympatry is that the phenotypes favored by reinforcement can differ from those favored by intraspecific mate choice [11] in regions of allopatry, producing direct fitness costs to enhanced species recognition in areas where hybridization does not occur. In such cases, species-recognition systems, either the courtship signals, mating preferences, or both, will evolve at the expense of those typically involved in mate choice within species [12], [13], producing patterns of enhanced interspecific mate recognition in individuals from populations in regions of sympatry and perhaps more discriminating intraspecific mate preferences in individuals from regions of allopatry. A related possibility is that enhanced species recognition imposes fitness costs indirectly, which limits enhanced recognition to regions of sympatry [14]. For example, the evolution of other traits related to mate choice, including elaborate courtship behaviors (e.g. zig-zag and rolling dances [15]) or investment in specialized signaling or sensory structures (e.g. fin size [16]), could enable enhanced species discrimination and thus avoid hybridization in regions of sympatry. However, absent the risk of hybridization, fitness decrements associated with such traits would select against their evolution or maintenance in regions of allopatry. Under either scenario, relaxation or loss of selection against hybridization - for example through shifts in species ranges that alter or eliminate regions of sympatry [17] - would favor the loss of such species recognition traits and favor a return to the phenotype expressed in regions of allopatry.

Alternatively, costs associated with greater species-recognition need not be invoked to explain the geographic patterns of interspecific mate discrimination. Instead, the traits involved in prezygotic isolation could be effectively neutral in allopatry, and thus mate preference among conspecific populations may be free to diversify via genetic drift or population-specific patterns of sexual selection [18], [19]. This would produce a geographic pattern where individuals from populations in regions of sympatry would exhibit relatively strong, uniform species-discrimination abilities whereas individuals from regions of allopatry would possess weaker discrimination abilities and stronger, population-specific intraspecific mate preferences [18].

In both cases outlined above, interspecific mate discrimination may be reduced or even absent in regions of allopatry because alleles for species discrimination are not beneficial in the absence of selection to avoid hybridization [17]. Establishing which of these scenarios occur in nature can inform our understanding of the processes maintaining patterns of mate discrimination both in sympatry and in allopatry, processes which in turn contribute to the generation and maintenance of barriers between closely related species.

For a number of reasons, it is challenging to study the response of traits involved in enhanced species recognition to relaxed selection against hybridization. In nature, the degree of contact between species frequently grades from high in regions of true syntopy and lessens with distance as sympatry gives way to allopatry. Moreover, the size, location and intensity of this gradient may fluctuate over time. Together, this means that it can be difficult to establish precisely when and the degree to which evolutionarily relevant contact between taxa ceases and true allopatry begins. In addition, competition between sibling species within the region of sympatry can lead to divergent selection on other traits, particularly those associated with food acquisition [3], [20]–[22]. The resulting ecological character displacement can be confounded with, or even mistaken for, reproductive character displacement associated with selection for enhanced species discrimination [3], [12]. All this, combined with gene flow between regions of sympatry and allopatry, can complicate empirical investigation of the evolutionary fate of enhanced species recognition [6], [23], [24]. One solution to these problems is to study populations taken from regions of sympatry and maintained in experimental allopatry [25]. Because naturally occurring variation among populations can persist during routine care and maintenance of laboratory cultures [26], single-species collections from nature represent a rich resource for the experimental study of species-recognition systems and how they might affect the maintenance of species boundaries.

The behavior and genetics of mate choice, speciation, and reinforcement have been particularly well studied in *Drosophila pseudoobscura* and *D. persimilis*, and reinforcement in this sibling species pair has been well documented in several independent studies [27]–[34]. Starting with Dobzhansky's pioneering work [1], [27], [35], collections of these species have been made from geographic populations within regions of allopatry and sympatry and maintained as laboratory isolates. For collections made from within the region of sympatry, these isolates represent populations that have evolved heightened species recognition abilities in nature [2] that were placed into and maintained in experimental allopatry. Hence, these historical collections offer a unique resource that can be used to study the evolutionary fate of enhanced species recognition once selection against hybridization has been removed.

Here, we explore variation in the strength of species recognition between *D. pseudoobscura* and *D. persimilis* to uncover the evolutionary fate of enhanced species discrimination associated with reinforcement. Using historical collections maintained in experimental allopatry, we examine differences in propensity to hybridize between populations collected from within the regions of sympatry and allopatry. Our experimental design is powerful because we assay populations that have been maintained in experimental allopatry for 10 to 60 years (up to ∼880 generations), allowing inferences to be drawn about the fate of species discrimination across multiple time points. Additionally, our design enables us to examine changes in mate discrimination resulting from changes in traits related to male courtship and/or female preferences. If enhanced species discrimination is costly, either through direct selection against female discrimination or through selection on traits linked to female choice, we predict that *D. pseudoobscura* females originating from regions of sympatry with *D. persimilis* will become less discriminating with increasing time since isolation. However, if there is no cost to enhanced species recognition, we expect that populations collected from the regions of sympatry will have higher species discrimination abilities than their allopatric counterparts.

## Methods

### Study system

**Figure 1:**
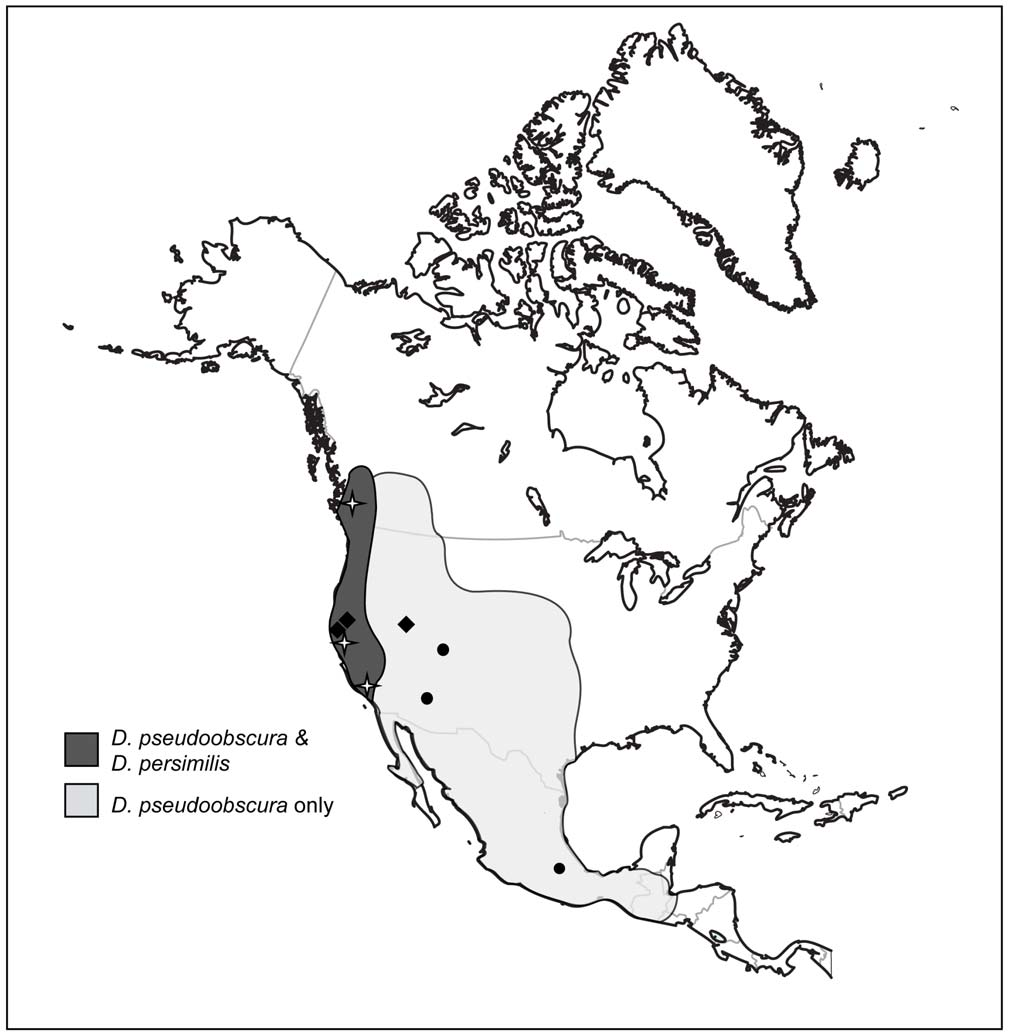
Species ranges and localities. The distribution of *Drosophila pseudoobscura* and *D. persimilis* [57]. Localities for allopatric populations of *D. pseudoobscura* are shown in black circles and sympatric populations in black diamonds. Localities for *D. persimilis* are indicated by open stars.

*Drosophila pseudoobscura* is a wide-ranging species, occurring from southwestern Canada, through the western United States, and into Mexico (Figure 1). The range of *D. persimilis* is contained exclusively within the northern Pacific coastal range of *D. pseudoobscura* (Figure 1). These sibling species diverged ∼850,000 to 500,000 years ago [36], [37]. Hybrid matings occur naturally at a low frequency in the wild [38], [39], resulting in hybrid male sterility [40], [41]. Males court females of both species indiscriminately and mate choice is driven largely by female preference for conspecific males [2], [42]. Documented patterns of species discrimination within *D. pseudoobscura* indicate that reinforcement is occurring; preference for conspecific males is greater in females from populations where *D. pseudoobscura* and *D. persimilis* are sympatric than in females from allopatric populations, and species discrimination ability varies in females among sympatric populations [31].

### Lineages and rearing conditions

Multiple lineages from both sympatric and allopatric populations of *D. pseudoobscura* and *D. persimilis* were obtained from the Drosophila Species Stock Center for each of three time intervals of collection: 1950s, 1990s, and 2000s (Table 1; strains Mather 17 and Mather 32 were kindly provided by M. Noor). These intervals were chosen because they contained *D. persimilis* and *D. pseudoobscura* population collections from within the regions of sympatry and allopatry. Given these criteria and culture availability, we were restricted in the final composition of our sample populations (Figure 1, Table 1, and see below). Where possible, isolates were chosen from similar geographic localities across time periods. We were able to use flies from the same sympatric population (Mather, California) at each of the three collection times. All lineages had been maintained in culture at least 1 year prior to acquisition. The experiments were conducted in late spring and early summer of 2009, thus the majority of 2000s lineages represent a virtually contemporary collection. Flies were raised on a sucrose-yeast-agar diet and maintained on a 12∶12 light cycle at 20°C and 75% humidity. Flies were reared in food vials at moderate larval density with relatively little variation in subsequent adult body size.

**Table 1:** Species lineages.

Species	Lineage	Population	Location	Year of Collection
*D. persimilis*	111.01	-	Quesnel, British Columbia	1958
	111.46	-	Mount St. Helena, California	1997
	111.48	-	Mount St. Helena, California	1997
	111.5	-	Santa Cruz Island, California	2004
	111.51	-	Santa Cruz Island, California	2004
*D. pseudoobscura*	121.88	allopatric	Edo de Mexico, Mexico	1958
	121.89	allopatric	Edo de Mexico, Mexico	1958
	121.94	allopatric	Mesa Verde, Colorado	1996
	121.151	allopatric	Flagstaff, Arizona	1993
	121.15	allopatric	Organ Pipe Monument, Arizona	2007
	121.153	allopatric	Wilcox, Arizona	2007
	121.41	sympatric	Mather, California	1959
	121.42	sympatric	Mather, California	1959
	121.64	sympatric	Mather, California	1959
	Mather 17	sympatric	Mather, California	1997
	Mather 32	sympatric	Mather, California	1997
	121.103	sympatric	Mather, California	2001
	121.104	sympatric	Mather, California	2001
	121.148	sympatric	San Francisco, California	2006
	121.155	sympatric	Eugene, Oregon	2006

Fly lineages used in outcrossing and subsequent courtship trials. Lineages grouped in boxes were crossed to generate an outcrossed population from which flies were collected for experiment. 111.01 was not outcrossed as no additional population from that collection year was available. All lines were from the Drosophila Species Stock Center, except Mather 17 and 32 (provided by M. Noor).

### Crosses and Mating Assays

To reduce potential inbreeding depression, lineages from the same species, time point, and where possible, populations were crossed in both directions (Table 1) [2], [25], [31], [32], [43]. When crosses from the same population were not possible, crosses were made using the nearest locality from that time period. Within 4 hours of eclosion, unmated progeny from these crosses were anesthetized with CO_2_, sorted by sex and placed into single-sex vials with food for 9 days. At least 1 day prior to the experiment, males and females were aspirated into individual food vials to reduce courtship inhibition resulting from crowding [44]. Mating assays were conducted 11 days post-eclosion, during the first three hours of light cycle. For the trials, an individual female was placed in the male vial and the cotton plug pushed down such that approximately 2.5 cm (∼10 cm^3^) of space remained in which flies could interact [45], (Noor pers. comm.). On any given day, all possible mating trial combinations (Table 2) were conducted and trials were randomized for time since collection and pair direction both within and across days. This “no-choice” design employs well-established protocols for this species pair [2], [30], [45], except that in our case flies were aged an additional 4 days to improve overall mating success (data not shown). All assays were conducted blind; the observer did not know whether crosses were conspecific vs. heterospecific, sympatric vs. allopatric, or the year of collection.

**Table 2:** Latency data.

A	1950s		1990s		2000s	
	N	Latency	N	Latency	N	Latency
♂ persimilis X ♀ persimilis	86	54 (50)	100	61 (60)	95	73 (65)
♂ pseudoobscura X ♀ pseudoobscura	100	46 (51)	99	40 (50)	101	42 (43)
♂ pseudoobscura(S) X ♀ persimilis	202	46 (56)	197	57 (59)	198	48 (51)
					198	43 (51)
♂ pseudoobscura(A) X ♀ persimilis	200	49 (54)	201	60 (66)	200	44 (52)
♂ persimilis X ♀ pseudoobscura(S)	197	62 (55)	196	56 (51)	198	77 (66)
					198	62 (54)
♂ persimilis X ♀ pseudoobscura(A)	196	61 (58)	199	72 (62)	195	63 (54)

Sample size and average courtship latency in seconds for each type of conspecific and heterospecific pairing for each of the three collection time points. Standard deviations shown in parentheses.

Trials were observed for up to 5 minutes to determine the time of the onset of courtship, typically identified by male wing vibration or rarely a copulation attempt [46], followed by an additional 5 minute observation. Time to initiate courtship (courtship latency), number of copulation attempts and time until successful copulation were recorded using a custom software program (FlyMate, available by request from EMM). Each fly was used only once [32]. Following mating trials, food vials were cleaned with ethanol and reused in subsequent mating trials on later days.

### Analysis and Interpretation

We made multiple comparisons to evaluate changes in species discrimination patterns over time. We compared courtship and mating characteristics for conspecific versus heterospecific crosses to confirm that male court females of either species indiscriminately and that females preferentially mate with conspecifc partners over heterospecific males. For conspecific *D. pseudoobscura* trials, pairings were constructed equally from allopatric and sympatric populations. We also compared courtship and mating variation between sympatric and allopatric populations in the heterospecific crosses, as well as among the three collection times (1950s, 1990s, and 2000s). Analyses include only trials where at least one copulation attempt occurred; however, including males that performed courtship song but did not attempt copulation did not affect our results (analyses not shown). A contingency table chi-square test was used to assess two indices of pair mating success, the number of successful copulations (copulation that lasted more than 60 seconds) [32], and the Noor Score, a metric of mating success in which a score of 2 is assigned for successful copulation within two attempts, 1 is assigned to all other successful copulations, and 0 is assigned to unsuccessful pairings [30]. The effect of cross type (e.g. conspecific/heterospecific, allopatry/sympatry, collection time) on courtship latency and number of copulation attempts was analyzed by a series of six individual one-way ANOVAs. Subsequent post-hoc pair wise comparisons were made using Tukey-Kramer tests [47], [48]. For all crosses, Clopper-Pearson exact binomial confidence intervals were calculated using JavaStat [49], [50].

## Results

We observed 3,949 courtship trials of which 3,356 (85%) contained at least one copulation attempt (Table 2). Neither time since isolation nor cross type (conspecific versus heterospecific) affected the occurrence of at least one copulation attempt (χ^2^ = 5.13, df = 2, p>0.05; χ^2^ = 0.85, df = 1, p>0.05 respectively). However, *D. persimilis* males were significantly less likely to attempt at least one copulation (regardless of the female they were paired with) than their congeneric counterparts (χ^2^ = 83.164, df = 1, p<0.0001) and copulation attempts were less frequent with *D. pseudoobscura* females, regardless of the identity of the courting male (χ^2^ = 17.668, df = 1, p<0.0001).

As predicted, there was no difference in courtship latency for crosses involving males courting conspecific or heterospecific females (F_1,3354_ = 3.21 p = 0.073; Table 2) although across all pairings, *D. pseudoobscura* males courted more quickly and had more copulation attempts than *D. persimilis* males (mean ± SE; Latency: *D. persimilis* 64.38±1.38 sec, *D. pseudoobscura* 48.44±1.36 sec, F_1,3354_ = 67.51, p<0.0001, Table 2; Copulation Attempts: *D. persimilis* 3.56±0.094, *D. pseudoobscura* 5.28±0.094, F_1,3353_ = 165.99, p<0.0001; Table 3). There was no change in this general pattern when each of the collection time points was examined individually (Table 2; Table 3).

**Table 3:** Copulation data.

	1950s	1990s	2000s
	Copulation Attempts	Copulation Attempts	Copulation Attempts
M persimilis X F persimilis	1.81 (1.49)	1.34 (0.79)	1.44 (2.33)
M pseudoobscura X F pseudoobscura	1.30 (0.81)	1.61 (1.66)	1.70 (1.55)
	6.52 (3.91)	4.17 (3.67)	6.97 (5.32)
			8.78 (5.95)
M pseudoobscura(A) X F persimilis	6.12 (3.99)	4.32 (3.49)	5.70 (4.29)
M persimilis X F pseudoobscura(S)	3.18 (2.13)	4.86 (3.78)	2.78 (1.98)
			4.09 (2.82)
M persimilis X F pseudoobscura(A)	3.54 (2.39)	5.34 (3.19)	4.02 (2.87)

Number of copulation attempts for each type of conspecific and heterospecific pairing for each of the three collection time points. Standard deviations shown in parentheses.

**Figure 2:**
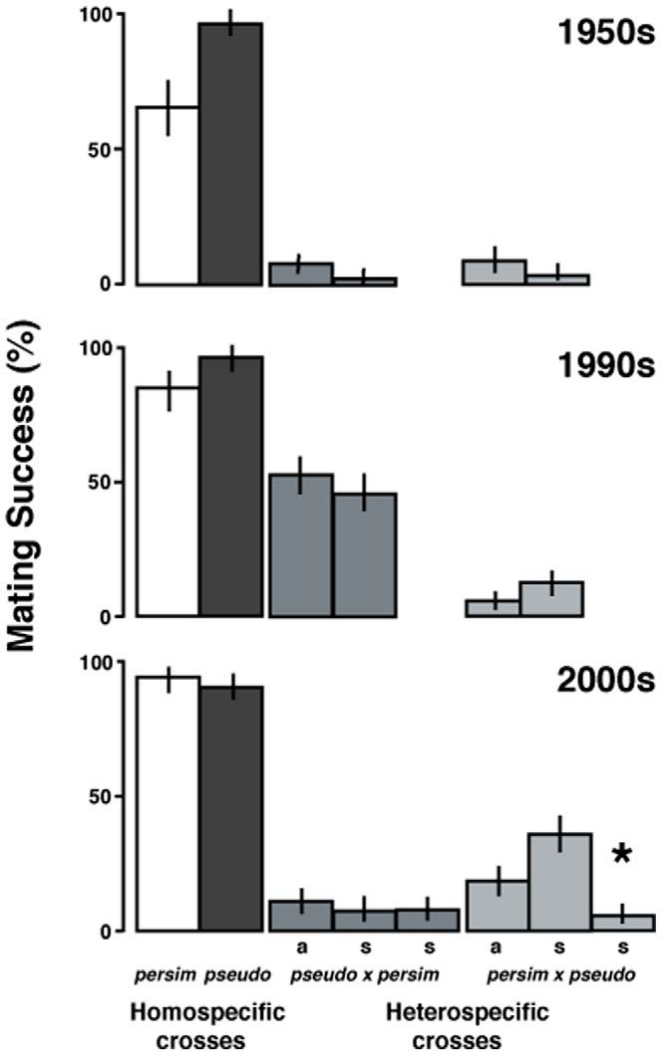
Copulation success. Copulation success rates for conspecific and both directions of sympatric (s) and allopatric (a) heterospecific pairings (male×female) between *D. pseudoobscura* (pseudo) and *D. persimilis* (persim). Panel is divided by the time of lineage collection. Clopper-Pearson exact binomial confidence intervals are given for each cross type and statistically significant pattern consistent with reinforcement is indicated with a star.

Mating success for conspecific pairings ranged from 72% to 95%. *D. persimilis* mated more readily with conspecifics as time since isolation decreased, whereas *D. pseudoobscura* exhibited the opposite pattern (Figure 2). Heterospecific pairings were less successful (2–51%). If enhanced species recognition is costly, then we expect species discrimination ability to be negatively correlated with time since isolation, however the pattern of successful copulations varied among collection time points and pairing type. Specifically, our data provide mixed support for the predicted pattern of stronger species discrimination in heterospecific pairings of *D. pseudoobscura* females from regions of sympatry relative to those from regions of allopatry (Figure 2, Table 4). For populations isolated in the 2000s, the results were split; the sympatric Mather population mated with *D. persimilis* at significantly higher levels than the allopatric population. In contrast, the non-Mather sympatric population exhibited a significant pattern consistent with a history of reinforcement. For the historically isolated populations, the 1990s sympatric population differed significantly from the allopatric population in the direction opposite of that expected under reinforcement, and the 1950s populations were indistinguishable in their species discrimination ability. Results were similar using the Noor Score alternative metric of mating success (Table 4).

**Table 4:** Analysis of mating success across collection times.

A					
Collection	N	df	-Log Likelihood	Chi Square	P-value
1950s	393	1	2.137	4.274	0.0387
1990s	395	1	3.187	6.373	0.0116*
2000s					
Mather	393	1	7.216	14.433	0.0001*
Non-Mather	393	1	7.325	14.65	0.0001*

B					
Collection	N	df	-Log Likelihood	Chi Square	P-value
1950s	393	2	2.522	5.044	0.083
1990s	395	2	3.208	6.416	0.404
2000s					
Mather	393	2	8.326	16.651	0.0002*
Non-Mather	393	2	7.46	14.919	0.0006

Analysis of variation in (A) mating success and (B) Noor Score between sympatric and allopatric populations of *D. pseudoobscura* females paired with *D. persimilis* males from each of the three collection times. P-values in bold indicate values significant after sequential bonferroni correction, asterisk indicates pattern in the opposite direction from that expected under reinforcement.

## Discussion

Studying the evolutionary fate of enhanced species discrimination provides insight into the processes and mate choice behaviors generating and maintaining species barriers, as well as the pliability of the barriers themselves. Freed from the constraints imposed by strong selection against hybridization, it is unclear how species-level mating preferences will respond to relaxed selection. Historical, single-species collections provide a potentially powerful experimental system with which to track changes in mating preference patterns over time, perhaps particularly so when applied to studies of the evolutionary fate of enhanced species discrimination following the loss of natural selection against hybridization. Using historical collections maintained in experimental allopatry for hundreds of generations, we found considerable variation in several aspects of mating propensity within and across collection times and cross types. Despite this variation, however, some important trends are evident. In general, we found lower levels of successful mating across all pairing types relative to other studies with this system [2], [45]. And, although heterospecific matings occurred at relatively low frequency regardless of collection time or the populations paired, only the lineages experimentally isolated most recently exhibited enhanced mate discrimination in a pattern consistent with being formed by reinforcement. This suggests that enhanced species discrimination may decay readily once selection against hybridization is removed. Below we attempt to determine what might be responsible for these patterns.

**Figure 3:**
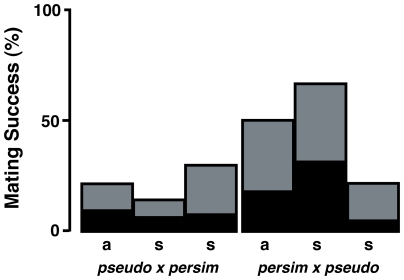
Diet effects on mating success. Copulation success rates for both directions of sympatric (s) and allopatric (a) heterospecific pairings (male×female) between *D. pseudoobscura* (pseudo) and *D. persimilis* (persim) from the 2000s time point reared on sucrose only diet (black bars) and sucrose+dextrose diet (gray bars).

Larval diet can influence the degree of species discrimination in other Drosophilids [51], and so we performed mating trials to determine if our observed pattern of generally lower levels of mating success across all time points could be explained by diet. To explore fully this possibility, we would ideally conduct all mating trials using larvae from all populations reared on diets used in earlier studies and test for effects of larval diet on species discrimination. While the exact diet used in the earliest research is not known for certain [27], two media types were prevalent including Kalmus media [52] and Spassky Cream of wheat media [53]. We did not explore the effects of these potential media. However, we reared approximately 25 crosses of each pair type for the 2000s time point on a food recipe used in several other recent studies [32], [54], (Noor pers. comm.), which differed from our recipe in that it contained dextrose in addition to sucrose (Table S1). All pairings exhibited higher mating success on this enriched diet (Figure 3). Although statistically significant differences in the relative proportions of successful matings are seen compared to a standard average increase (χ^2^ = 17.29, df = 5, p = 0.00398), much of this was driven by the Mather population (Mather 2000s discussed further below); excluding the Mather population upheld the relative levels of species discrimination among groups regardless of larval diet (χ^2^ = 6.739, df = 3, p = 0.0807). Thus, while larval diet was responsible for the pattern of overall reduced mating success in the current study relative to earlier work with this system [2], [32], [45], we reject diet as being responsible for the observed variation in mating success among populations and time points.

Differences in the number of successful copulations (i.e., copulations lasting at least 60 seconds) between heterospecific and conspecific crosses were not a function of variation in male courtship intensity. Courtship latency was similar across cross types and time periods and was consistent with other studies [2], [42]. *Drosophila pseudoobscura* males court on average 16 seconds faster and have 2 more copulation attempts than males of *D. persimilis*. Although such interspecific differences in courtship intensity could have important evolutionary consequences if females select between directly competing males, such scenarios are likely limited in nature [31].

The absence of a role for male courtship intensity or persistent enhanced mate discrimination following the establishment of experimental allopatry indicates that enhanced female choosiness for conspecific males may carry some cost and thus be selected against in the absence of reinforcement. This likely suggests that, in this case selection for enhanced interspecific mate discrimination, selection may have seized upon the same traits involved in intraspecific sexual selection. Although the exact targets of selection are unknown, possibilities include cuticular hydrocarbon profiles [13] or courtship song pattern [29].

Our results suggest a role for selection against enhanced species discrimination in experimental allopatry, however, the observed variation in species discrimination among experimental lineages indicates that drift may also be an important factor. Within collection time periods, variation in mating success among the sympatric populations of *D. pseudoobscura* reflects a genetic basis for variation in species discrimination; considerable standing variation in species discrimination exists within both sympatric and allopatric populations [32], [45]. The high levels of mating success exhibited by the Mather 2000 lineages is unusual in that these flies mated more readily than even the allopatric populations of *D. pseudoobscura,* and at a higher frequency than any other heterospecific cross type in our study. Indeed, the mating propensity we observed exceeds the levels reported for this population in other studies, particularly after accounting for the effects of diet (e.g., 67% success on comparable sucrose-dextrose food recipe, Figure 3; compared to maximum values of 10–45% [45]; 16.67% [43]; 15.6–36.5% [2]; 30% [30]; 0–4.26% [31]; 8.6–48% [32]). As inbred lineages demonstrate higher variation in mating success than outbred populations [32], the strong propensity to mate with heterospecifics exhibited by the Mather 2000 lineage may indicate that it suffers from low genetic diversity. Preliminary genetic analysis of the two lineages that were crossed to create the Mather 2000 lineage for this experiment found that each possessed the same rare allele at a locus on the XL chromosome (Noor pers. comm.), suggesting that the lineages used in this study established from those stocks had lower genetic diversity than other lineages used in the study. Thus, low genetic diversity at the loci contributing to species discrimination may help explain the relatively indiscriminant mating pattern exhibited by this lineage.

Our research highlights the interplay between populations in studying mate preferences generally, and establishing the presence or absence of patterns consistent with reinforcement. Studies assessing the presence or absence of reinforcement do so based on enhanced mate discrimination in sympatric populations relative to allopatric ones. This comparison inherently ties experimental outcomes to not just the sympatric population of interest, but also to the allopatric reference population. Thus, changes in the relative choosiness of an allopatric population can determine whether or not a pattern consistent with reinforcement is seen, regardless of choosiness of the sympatric population.

Although our experimental approach offers some advantages over traditional comparative methods that draw on extant natural populations, it brings some complications of its own. First, while being maintained in single-population isolates in the laboratory, flies experience relaxed selection for enhanced mate discrimination and are simultaneously subjected to novel selection on other traits related to lab adaptation [25]. Consequently, selection may occur on secondary traits correlated with the focal traits, changing the focal trait value even in the absence of direct selection on species discrimination. In addition, founder effects and small population sizes coupled with occasional bottlenecks may increase the importance of drift, affecting mating preferences and levels of discrimination between lineages within and between species in unpredictable ways as similar effects have been seen in other traits [26]. Cycles of founder-flush-bottlenecking can produce ethological isolation between populations and inbreeding depression can reduce the mating propensity even with members of the same population [55], [56]. Hence, negative results discovered using our approach must be interpreted with caution.

In conclusion, historical laboratory collections offer a unique and powerful experimental tool to address questions regarding the evolutionary fate of reinforcement-driven enhanced interspecific mate discrimination once selection against hybridization is relaxed. While our experimental design is powerful, limited numbers of stocks limited the number of populations available to test our hypotheses. More independent populations, for example, would allow for a more rigorous testing of the role of drift or selection in producing our observed patterns; unfortunately, such additional replicated populations are not available. This highlights one of the key values of preserving historical cultures and calls for regular and repeated sampling from the same localities; deposition and culture of lineages in this manner will preserve biological resources – time capsules – which are likely to be of increased utility as species distributions change. Our finding of generally reduced species discrimination with time spent in experimental allopatry likely suggests that intraspecific sexual selection may alter mating preferences in the absence of selection against hybridization; however, drift likely moderates mating preferences as well. The patterns of mating preferences observed for any population allow us to glimpse the many selective forces acting on the pliability of the species barrier across both space and time.

## Supporting Information

Table S1 **Food recipes.** Food recipes used in examining mating success based on diet based on a 1-liter recipe.
(DOC)
