# Supplementary material for: Time in a Bottle: The Evolutionary Fate of Species Discrimination in Sibling Drosophila Species
Source: PLoS One. 2012 Feb 27;7(2):e31759. doi: 10.1371/journal.pone.0031759 (PMC3288057; doi:10.1371/journal.pone.0031759)
Supplement: Table S1 — Food recipes. Food recipes used in examining mating success based on diet based on a 1-liter recipe. (DOC) [file pone.0031759.s001.doc]

| **Ingredient** | **Standard Diet** | **Noor Recipe** |
| --- | --- | --- |
| Water | 875mL | 1000mL |
| Agar | 7.5g | 14g |
| Yeast | 22.5g | 100g |
| Tegosept | 2g | 2g |
| Sucrose | 11g | 50g |
| Dextrose | 0g | 50g |
| Cornmeal | 52.6g | 0g |
| Propionic Acid | 5.5mL | 4.18mL |
| Phosphoric Acid | 0mL | 0.415mL |
